# Exposure to Di-2-ethylhexyl Phthalate and Benign Prostatic Hyperplasia, NHANES 2001-2008

**DOI:** 10.3389/fendo.2021.804457

**Published:** 2022-01-13

**Authors:** Luchen Yang, Zhenghuan Liu, Zhufeng Peng, Pan Song, Jing Zhou, Linchun Wang, Junhao Chen, Qiang Dong

**Affiliations:** Department of Urology, Institute of Urology, West China Hospital, Sichuan University, Chengdu, China

**Keywords:** benign prostatic hyperplasia (BPH), di-(2-ethylhexyl) phthalate (DEHP), NHANES, phthalates, biphasic effects

## Abstract

30% of men suffer from benign prostatic hyperplasia (BPH) worldwide. As one of the most important members of Phthalate esters, previous studies suggested ubiquitous Di-(2-ethylhexyl) phthalate (DEHP) exposure is associated with such male disorders by interfering with endocrine system, however, little is known about the association between DEHP exposure and BPH. The objective of this study was to study the potential association by the 2001–2008 National Health and Nutrition Examination Survey (NHANES) data. The data was collected, and multiple logistic regression was adapted to measure the association. The concentrations of DEHP (∑DEHP) were calculated by each metabolite and split into quartiles for analysis. Results showed that the odds ratio (OR) decreased with increased ∑DEHP concentration. In the crude model, the OR for the second quartile (OR = 1.60, 95%CI [1.24, 2.07]) was obviously higher compared with the lowest quartile. However, the OR for the highest quartile (OR = 0.55, 95%CI [0.44,0.69]) was lower than that for the third quartile (OR = 0.77, 95%CI [0.61, 0.97]), and the OR for the third and the highest quartile were significantly lower than that of the lowest quartile, which suggested biphasic effects of DEHP based on concentration. The results showed the same trend after adjusting confounding factors. The study suggested that the DEHP exposure is associated with DEHP, and the results adds limited evidence to study this topic, however, further researches are needed to determine if the status of BPH can be changed by controlling DEHP exposure.

## Introduction

Di-(2-ethylhexyl) phthalate (DEHP), as one of the most widely used plasticizers, is a recognized endocrine-disrupting compound, and it is ubiquitous in the environment because of its non-covalent and highly hydrophobic properties which allow DEHP to escape from plastics easily (1). Simultaneously, DEHP can enter into body by various ways including inhalation, ingestion, and dermal exposure (2). More than 300 μg/kg concentration of DEHP has been found in daily diet such as meat and lipid-rich products (3). After uptake into the human body, the de-esterifiction of DEHP occurs under the influence of esterase or lipase, and DEHP transforms into its metabolites, such as MEHP, MEOHP, MEHHP and MECCP. Eventually, these toxicants are mainly eliminated in urine (4). Previous studies suggested that DEHP and its metabolites can interfere with sex hormone levels in the body and have adverse effects on the male reproductive system, including decreased testosterone levels, sperm production, increased risk of infertility, hypospadias, cryptorchidism, and testicular cancer (5). Simultaneously, DEHP has been reported to be associated with prostate disorders in animal experiments (6).

As one of the most important male reproductive glands, prostate is mainly responsible for urinary control, sexual function and reproduction. The major disorders of prostate include benign prostatic hyperplasia (BPH), prostate cancer (PCa), and prostatitis (7). BPH is the most common benign disease causing voiding disorder in middle-aged and elderly men (8). The disorder is characterized by histological hyperplasia of prostate stroma and glandular components, anatomical enlargement, urodynamic bladder outlet obstruction (BOO) and the lower urinary tract symptoms (LUTS). The incidence rate of BPH increases with age, usually after 40 years old (9). The specific mechanism of BPH is unclear. As a multifactorial disease, age, the interaction of androgen and estrogen, growth factors, inflammatory cells, genetic factors and lifestyle may be potential factors related to the development of BPH (8). Previous experimental studies (6) have suggested that exposure to DEHP is associated with BPH, and DEHP has potential to stimulate cell proliferation by regulating the expression of receptors such as androgen and estrogen receptor. Simultaneously, DEHP is able to enlarge prostate under the effects of interfering with the sex hormones and causing oxidative stress. However, it is unclear whether DEHP is associated with the development of BPH in the population. And, most animal and human studies of DEHP mainly concentrate on the male reproductive functions, little is known about the possible effects on BPH. Hence, under the objective of assessing the potential association between DEHP exposure and BPH, our study adopted a nationally representative sample from the 2001–2008 National Health and Nutrition Examination Survey (NHANES) data.

## Materials and Methods

### Study Population

We used data from four cycles (2001–2002, 2003-2004, 2005-2006 and 2007-2008) of National Health and Nutrition Examination Survey (NHANES) to study the association between urinary DEHP metabolites and BPH. Conducted by the CDC’s National Center for Health Statistics, NHANES collects data that represent the health and nutritional status of the civilian, non-institutionalized U.S. population. As a national, cross-sectional survey, NHANES structures a series of physical examinations, laboratory tests and questionnaires to acquire information that is sampling-probability based. Simultaneously, these data are representative of the U.S, and all participants have signed informed consent. Data used are publicly accessible on NHANES web site, and the program was approved by the NCHS Research Ethics Review Board (ERB).

In our study, men who participated in the prostate condition questionnaire were considered. Of the 9544 candidates whose data regarding prostate condition was available, we further set up a series of exclusion criteria: 1. other prostate problems such as prostate cancer and prostatitis, or other questions such as “Bladder feels empty after urinating”; 2. urinary DEHP metabolite data lost; 3. creatinine concentrations less than 30 or more than 300 mg/dL (10); 4. missing information on age, race/ethnicity. The exclusion criteria eventually resulted in 280 participants. Previous studies have mentioned the creatinine-related biases when study phthalate exposures using urinary metabolites levels, and to avoid the effects of urine dilution, creatinine-corrected were applied for the levels of each metabolite (11). The data of study population characteristics by BPH status are detailed in **Table 1**.

**Table 1:** Study population characteristics by BPH status; NHANES 2001–2008.

	BPH status	
	No	Yes
Population	72	208
Urine Creatinine (mg/dl)	137.4	119.9
AGE (years)	75.1	67.7
BMI (kg/m2)	28.4	29.1
Serum Testosterone (ng/dl)	4.5	4.4
Race/ethnicity(%)		
Mexican American	13.2	7.9
Other Hispanic	4.2	4.9
Non-Hispanic White	63.5	72.5
Non-Hispanic Black	16.2	12.8
Other Race	2.9	2
Education(%)		
Less than high school	44.1	22.6
High School Grad/GED or Equivalent	15.6	23.7
More than high school	40.3	53.8
Marital Status (%)		
Married	70.9	76.6
Single	24.9	21.6
Living with a partner	4.1	1.8
Hypertension Status (%)		
Yes	57.9	56.3
No	42.1	43.7
Diabetes Status(%)		
Yes	17.9	16.1
No	82.1	83.9
Coronary Heart Disease Status (%)		
Yes	11.6	15.2
No	88.4	84.8
Smoke at Least 100 Cigarettes in Life		
Yes	72.1	67.7
No	27.9	32.3
∑2.3e at Least 100		
Lowest Quartile	22.3	25.6
Second Quartile	15.4	28.1
Third Quartile	27.7	24.4
Highest Quartile	34.6	21.9

### DEHP Metabolite Measurements

One-third participants were randomly selected for phthalates metabolite measurements in NHANES. In our study, four major DEHP metabolites were identified including mono (2-ethylhexyl) phthalate (MEHP), mono(2-ethyl-5-oxy-hexyl) phthalate (MEOHP), mono (2-ethyl-5-hydroxy-hexyl) phthalate (MEHHP), and mono (2-ethyl-5-carboxy-pentyl) phthalate (MECPP). These monoester metabolites have been shown as sensitive and representative of biomarkers reflecting DEHP exposure (12, 13). In our study, to avoid bias caused by results below the limit of detection (LOD), only the metabolites that were detected in at least 75% of the samples were considered, and all specimens were measured by solid phase extraction coupled on-line to high performance liquid chromatography and tandem mass spectrometry. The details were described in Centers for Disease Control and Prevention (CDC), Laboratory Procedure Manual: Phthalates and Phthalate Alternatives Metabolites. Simultaneously, specimen concentrations of the DEHP below the level of detection were calculated using LOD divided by the square root of two (14). Creatinine-corrected metabolites concentrations were adapted in our study through dividing the DEHP metabolite concentration by the urinary creatinine concentration with the objective of urine dilution correction, and our final unit was ng/mg crt (10).

### Measurement of BPH

Male participants were considered to be diagnosed with BPH when responding “yes” to the question “Enlargement was BPH”, and participants as follow were excluded: men who did not know the answer, refused to answer the question, or had a missing value.

### Statistical Analysis

Data was analyzed using the statistical packages R (The R Foundation; http://r-project.org; version 3.4.3) and EmpowerStats (www.empowerstats.com; X&Y solution inc). The complex sampling design and weights was adapted to our statistical analyses, which was recommended by NHANES, and P ≤ 0.05 was the level of significance. The concentration of ∑DEHP was calculated by dividing the molecular weight of each metabolite and summing, and then multiplying by the molecular weight of DEHP, as follow: {[MEHP × (1/278.34)] + [MEHHP × (1/294.34)] + [MEOHP × (1/292.33)] + [MECPP × (1/308.33)]} * 391 (15). In our study, natural log transformation of the ∑DEHP and metabolites has been conducted on each analyte prior to analysis because the strongly right skewed distribution. Next, we estimated the association of urinary ∑DEHP and BPH by the quartiles of ∑DEHP and metabolites based on the weighted distributions of these phthalate compounds in population study. Simultaneously, multi-variable logistic regression analysis was conducted, and odds ratios (ORs) and 95% confidence intervals (CIs) were calculated to evaluate the association. In our study, the confounding factors were identified based on previous literature or the variable in the final model changed the estimates of each metabolite exposure on BPH by more than 10%, and we constructed three main models: Model1 adjusted for no variable, which represented our crude model; Model2 adjusted for socio-demographic factors (age, race/ethnicity, education, marital status); Model3 adjusted for previous variables plus BMI, serum testosterone concentration, hypertension status, diabetes status, coronary heart disease status, and smoke condition.

## Results

**Table 1** has detailed the weighted distributions of study population (n = 280) characteristics of the total sample. Of those 280 men, 208 men (74.3%) reported BPH status, and 72 men (25.7%) without the history of BPH. The average age of men diagnosed with BPH was approximately 67.7 years old while 75.1 years old in men without the disorder. In our study, the majority of population was Non-Hispanic White, 72.5% in BPH men and 63.5% in non-BPH men respectively. Simultaneously, of all the samples, 76.6% of BPH and 70.9% of non- BPH men were married, and the mean serum testosterone concentration was 4.4 ng/dl in BPH men and 4.5 ng/dl in non-BPH men respectively. The geometric mean and median urinary concentrations of various metabolites by BPH status have been listed in **Table 2**. The distribution of ∑DEHP quartiles were divided as follow: <6.07 ng/mg crt represented the lowest quartile (Q1), 6.07-6.57 ng/mg crt represented the second quartile (Q2), 6.58-7.17 ng/mg crt represented the third quartile (Q3), and >7.17 ng/mg crt represented the highest quartile (Q4). In Q1, Q2, Q3, and Q4 of ∑DEHP, 22.3%, 15.4%, 27.7% and 34.6% men reported a history of BPH, respectively.

**Table 2:** Mean and Median of creatinine-corrected DEHP metabolites by BPH status; NHANES 2001–2008.

	BPH status			
	No n = 72		Yes n = 208	
	Mean (SE)	Median (IQR)	Mean (SE)	Median (IQR)
	(ng/mg crt)	(ng/mg crt)	(ng/mg crt)	(ng/mg crt)
MEHP	2.9 (1.1)	2.7 (2.1-3.7)	2.9 (1.0)	2.8 (2.2-3.5)
MEHHP	5.3 (1.0)	5.2 (4.6-5.9)	5.1 (1.0)	5.0 (4.5-5.7)
MEOHP	4.9 (1.0)	4.9 (4.3-5.4)	4.6 (1.0)	4.6 (4.0-5.1)
MECCP	5.8 (1.0)	5.9 (5.1-6.3)	5.6 (1.0)	5.5 (5.0-6.0)
∑DEHP	6.8 (1.0)	6.9 (6.2-7.4)	6.6 (1.0)	6.5 (6.0-7.1)

The results of the multivariable linear regression have been shown in **Table 3**. The results suggested a significant association between the ∑DEHP exposure and BPH. In the crude model (adjusted with no variable), the odds ratio (OR) for the second quartile (OR = 1.60, 95%CI [1.24, 2.07]) was obviously higher compared with the lowest quartile. However, the odds ratio (OR) for the highest quartile (OR = 0.55, 95%CI [0.44,0.69]) was lower than that for the third quartile (OR = 0.77, 95%CI [0.61, 0.97]), and the OR for the third and the highest quartile were significantly lower than that of the lowest quartile. A dose-response appeared, and compared with the lowest quartile, the OR decreased with increased ∑DEHP concentration. Simultaneously, the P for trend was <0.001 which suggested a statistically significance. We adjusted the socio-demographic factors (Model 2) and coupled with other factors (Model 3), the results were consistent with Model 1. Similar results were observed in the MEHHP, MECCP and MEOHP exposure. The association of MEHP exposure and BPH was analyzed, compared with the lowest quartile, the OR for the third quartile (OR = 2.24, 95%CI [1.75, 2.85]) in Model 1 was significantly higher, and the adjusted results were consistent.

**Table 3:** Association [OR (95% CI)] between creatinine-corrected DEHP metabolites and BPH; NHANES 2001–2008.

	Model 1	Model 2	Model 3
∑DEHP	280	280	280
Q1: <6.07 ng/mg crt	Referent	Referent	Referent
Q2: 6.07-6.57 ng/mg crt	1.60 (1.24, 2.07)	1.82 (1.36, 2.43)	1.73 (1.28, 2.35)
Q3: 6.58-7.17 ng/mg crt	0.77 (0.61, 0.97)	0.75 (0.58, 0.97)	0.76 (0.58, 0.99)
Q4: >7.17 ng/mg crt	0.55 (0.44, 0.69)	0.52 (0.40, 0.67)	0.51 (0.39, 0.67)
p trend	<0.001	<0.001	<0.001
MEHHP	280	280	
Q1: <4.53 ng/mg crt	Referent	Referent	Referent
Q2: 4.53-5.08 ng/mg crt	1.24 (0.97, 1.59)	1.40 (1.06, 1.86)	1.40 (1.04, 1.89)
Q3: 5.09-5.70 ng/mg crt	0.73 (0.58, 0.91)	0.60 (0.47, 0.78)	0.62 (0.48, 0.81)
Q4: >5.70 ng/mg crt	0.65 (0.52, 0.81)	0.59 (0.46, 0.76)	0.61 (0.47, 0.80)
p trend	<0.001	<0.001	<0.001
MEHP	280	280	280
Q1: <2.15 ng/mg crt	Referent	Referent	Referent
Q2: 2.15-2.81 ng/mg crt	1.24 (0.99, 1.55)	1.35 (1.05, 1.73)	1.27 (0.97, 1.67)
Q3: 2.82-3.50 ng/mg crt	2.24 (1.75, 2.85)	1.90 (1.45, 2.48)	1.93 (1.47, 2.54)
Q4: >3.50 ng/mg crt	1.05 (0.85, 1.30)	0.82 (0.64, 1.04)	0.84 (0.65, 1.09)
p trend	0.06	0.46	0.88
MEOHP	280	280	280
Q1: <4.03 ng/mg crt	Referent	Referent	Referent
Q2: 4.03-4.62 ng/mg crt	1.31 (1.01, 1.69)	1.54 (1.16, 2.05)	1.42 (1.05, 1.92)
Q3: 4.62-5.18 ng/mg crt	0.80 (0.63, 1.01)	0.83 (0.64, 1.08)	0.76 (0.58, 0.99)
Q4: >5.18 ng/mg crt	0.44 (0.35, 0.55)	0.39 (0.30, 0.50)	0.37 (0.28, 0.48)
p trend	<0.001	<0.001	<0.001
MECCP	280	280	280
Q1: <5.06 ng/mg crt	Referent	Referent	Referent
Q2: 5.06-5.57 ng/mg crt	1.22 (0.95, 1.57)	1.45 (1.10, 1.92)	1.37 (1.02, 1.82)
Q3: 5.58-6.16 ng/mg crt	1.10 (0.86, 1.40)	1.05 (0.80, 1.36)	0.97 (0.73, 1.27)
Q4: >6.16 ng/mg crt	0.45 (0.36, 0.57)	0.39 (0.30, 0.51)	0.40 (0.31, 0.53)
p trend	<0.001	<0.001	<0.001

Q, quartile. For each of the metabolites, Q1 is the reference. Model1 adjusted for no variable, which represented our crude model; Model2 adjusted for socio-demographic factors (age, race/ethnicity, education, marital status); Model3 adjusted for socio-demographic factors plus BMI, serum testosterone concentration, hypertension status, diabetes status, coronary heart disease status, and smoke condition.

## Discussion

In our study, a nationally representative crosssectional study was adapted to examine if the urinary DEHP was associated with BPH, and if the DEHP and metabolites could be biomarkers for BPH. The results indicated a clear association between DEHP exposure and BPH. Simultaneously, we observed a significant dose response trend that compared with the lowest quartile, the OR decreased with the increased DEHP concentration. Specially, the results showed the second quartile concentration of DEHP increased the risk of BPH (OR>1), while the third and highest concentration decreased the risk (OR<1), which suggested biphasic effects of DEHP based on concentration. Studies on this topic are scarce and findings are conflicting resulting from the complexity of DEHP exposure and BPH.

Prostate is one of the most important organs in male reproductive system, and BPH is the major cause of voiding disorder in middle-aged and elderly men (16). Although many pathogenic factors have been identified, the etiology of BPH is unknown. As a sex-steroids gland, enlarged prostate is associated with androgen/estrogen imbalance which could stimulate the proliferation of prostate cell during aging (17). Simultaneously, the balance of cell proliferation and cell death can be broken under the Interactions between growth factors and steroid hormones, which results in BPH (18). DEHP, as one of the most representative member of phthalates, has been shown to destroy the male reproductive system, and is positively associated with testicular dysgenesis syndrome, a collective disease, including hypospadias, cryptorchidism, male infertility, and testicular cancer (5). Simultaneously, the toxic effects of DEHP were exposure time- and dosage-dependent (19). DEHP usually interferes with the male reproductive system through oxidative stress (OS), DNA damage, hypothalamic–pituitary–gonadal axis (HPG axis) damage, and anti-androgenic or proestrogenic effects, which results in abnormal steroidogenesis, lower steroidogenic enzyme expression, and imbalance of cholesterol and lipid (20, 21). Previous experimental studies suggested that phthalates and their metabolites are associated with prostate cell proliferation in pca through affecting the production of androgen receptor (AR), estrogen receptor (ER), and interfering with gene pathway such as P38 (22–24). Simultaneously, DEHP might enlarge the prostate by OS. Wei Hsiang Chang et al. found that DEHP may induce oxidative stress, which cause inflammatory and, hence, result in benign prostatic hyperplasia (6). In the vivo, DEHP is rapidly metabolized to its metabolites, which can destroy mitochondrial function, and produce excessive reactive oxide species. Evaluated oxidative stress level can damage cellular DNA, which eventually generates the injury of cells and Inflammation (25, 26). The imbalance in the production and detoxification of OS is associated with the early stage of prostatitis and the development of BPH (27). Moreover, data from the Medical Therapy of Prostatic Symptoms study show that inflammation increased the BPH progression, and increased levels of inflammatory cells were also detected in the epithelial glands of human BPH tissues (28, 29). Although the lack of specific mechanisms and direct evidence, previous studies have indicated a possible association between DEHP and BPH, which is consistent with our results.

As an indispensable biomarker for BPH, testosterone could be affected by DEHP based on a large number of animal or *in vitro* experiments. However, previous studies suggested that the effect of DEHP on testosterone is biphasic. Ge et al. (30) suggested that the 10 mg/kg DEHP dose significantly advanced pubertal onset, whereas the 750 mg/kg DEHP dose delayed pubertal onset. Simultaneously, serum testosterone levels and seminal vesicle weights obviously increased in 10 mg/kg DEHP dose group, while serum T, testes and body weights significantly decreased in the 750 mg/kg dose group. Previous studies (31, 32) also suggested that rats exposed to 100 mg/Kg had higher testosterone while the levels of testosterone, FSH, and LH significantly decreased with 30-day exposure at doses of 900 mg/Kg. Consistent with the animal experiments, our study suggested a possibly biphasic effects of DEHP.

Several limitations in our study are as follow: First, the relatively small sample size hinders our further analysis. Additionally, causation of BPH cannot be identified from this analysis alone because of the cross-sectional study design. Furthermore, the manners of the measurement of BPH compounded the topic, which self-reported and measured in a historical way, and the onset of BPH was unknown. Finally, potential confounding factors that were either not involved in the study or unmeasured cannot be identified. Despite the limitations of our study, there are strengths. All participants and DEHP metabolites were included in our study, which covered an 8-year period. Moreover, to our knowledge, this is the first population-based study to examine the association of DEHP exposure and BPH among males.

In summary, our study suggested a clear association between DEHP exposure and BPH. Interestingly, we observed a significant dose response trend, which was the OR decreased with the increased DEHP concentration, and the results indicated possibly biphasic effects of DEHP based on concentration in male American population. Although our results add limited evidence to this topic, further researches are needed, not only because of the limitations of our study, but also because of the potential that the status of BPH can be changed by controlling DEHP exposure.

## Data Availability Statement

Publicly available datasets were analyzed in this study. This data can be found here: https://www.cdc.gov/nchs/nhanes/index.htm.

## Author Contributions

Study concept and design by LY. data acquisition by ZL. data analysis by LY and ZL. Data interpretation by ZP and PS. Manuscript drafting by LY. Critical revision of the manuscript for important intellectual content by LW, JZ, and JC. Study supervision by QD. All authors contributed to the article and approved the submitted version.

## Funding

This work was funded by the 135 project of West China Hospital of Sichuan University (ZY2016104); a Key Project of National Natural Science Foundation of China (No.8177060452);a Project of Science and Technology Department of Sichuan Province (2021YFS0117).

## Conflict of Interest

The authors declare that the research was conducted in the absence of any commercial or financial relationships that could be construed as a potential conflict of interest.

## Publisher’s Note

All claims expressed in this article are solely those of the authors and do not necessarily represent those of their affiliated organizations, or those of the publisher, the editors and the reviewers. Any product that may be evaluated in this article, or claim that may be made by its manufacturer, is not guaranteed or endorsed by the publisher.
